# Unraveling T-cell decoding strategy: a step forward

**DOI:** 10.1038/s44318-025-00645-4

**Published:** 2025-11-26

**Authors:** Philippe Robert, Pierre Bongrand

**Affiliations:** https://ror.org/055ymkj32 grid.503288.4 0000 0004 0385 5821 Lab. Adhesion & Inflammation, INSERM U1067/CNRS UMR7333/AMU U61, Marseille, France

**Keywords:** Immunology, Structural Biology

## Abstract

A new study in *The EMBO Journal* provides new insights into T-cell activation potency upon receptor-antigen interaction.

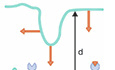

## Teaser

Elucidation of the mechanisms by which T-cells discriminate between self and foreign antigens has been a major challenge in immunology. In this issue of *The EMBO Journal*, Huhn and colleagues (Huhn et al, 2025) show that the affinity between soluble antigens and the widely-studied OT-I T-cell receptor is a good predictor of T-cell activation potency, which was previously thought to be determined by complex properties of the interactions between surface-bound antigens and receptors.

The outcome of pathological situations such as infectious or autoimmune diseases strongly depends on the immune system response to self or foreign antigens. This response is orchestrated by T-lymphocytes, and a key step is the response to antigens encountered as oligopeptides (p) bound to major histocompatibility complex (MHC) molecules borne by nearly all cell populations. The behavior of a T-cell has been long thought to be quantitatively and qualitatively determined by the interaction between its antigen receptors (TCR) and exposed pMHC complexes. In addition to its biological importance, antigen detection was shown to display remarkable features such as sensitivity and specificity, since a few or even a single pMHC complex can be detected on the surface of a cell exhibiting tens of thousands of fairly similar structures, as well as rapidity, since signaling events such as cytosolic calcium rise or phosphorylation may occur within a few seconds, while physiologically relevant cellular events such as arrest of a migrating cell or bulk cell spreading may be generated within a few seconds to one minute (Malissen and Bongrand, 2015). Importantly, a combination of sensitivity and rapidity is required to allow T-lymphocytes that are vastly outnumbered by surrounding cells to deal rapidly with infection. It is therefore not surprising that during the past three decades much work has been done to unravel the mechanisms of T-cell decision-making following antigen encounter.

During the 1990s, direct experimental evidence came to light supporting the hypothesis that the T-cell decision is tightly correlated to the quantitative properties of the TCR-pMHC binding, and particularly the dissociation rate, i.e., the frequency of bond rupture per second (Matsui et al, 1994). A mechanistic explanation for the T-cell discriminating power based on this model was rapidly elaborated: it was suggested that the TCR-pMHC complex triggers a series of biochemical reactions that is interrupted by dissociation of the pMHC ligand. The interaction might lead to inhibition if it is stopped before a limiting time point in the order of a few seconds (McKeithan, 1995). This hypothesis was termed the proofreading model since it was inspired by a model previously suggested to account for the accuracy of protein synthesis. This kinetic view is in line with the concept that the duration of TCR engagement might be a key activation parameter.

**Figure 1:**
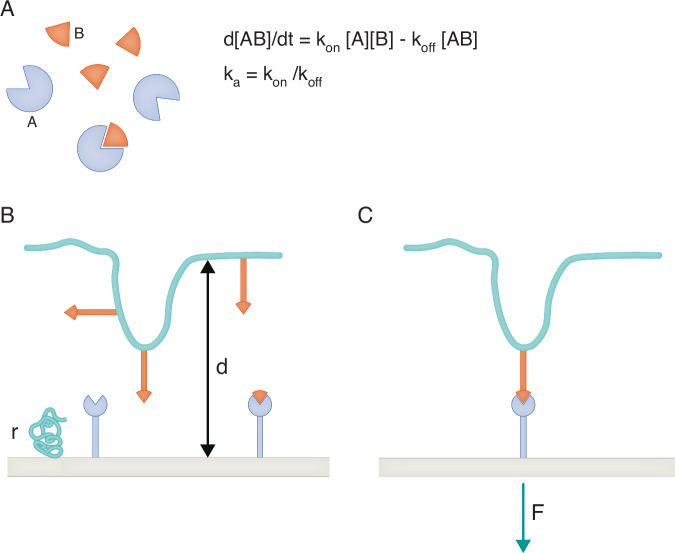
Difficulty of comparing 2D and 3D molecule association. (**A**) Conventional 3D molecular binding. Molecular encounters are well-described by standard diffusion equations. Molecular translation and rotation are weakly dependent on molecular size and shape. Bond rupture is driven by thermal fluctuations. The association rate (k_on_), dissociation rate (k_off_), and affinity constant (K_a_) are thus determined by molecular structure and generic mechanisms, they may thus be considered as intrinsic properties of interacting molecules A and B. (**B**) 2D association. When ligand and receptor molecules are bound to surfaces, the frequency of encounters is strongly dependent on the distance d beteween surfaces, surface roughness, flexibility of the anchors of binding sites that drive molecular contact frequency and relative molecule orientation, lateral diffusion of anchored molecules, and possible role of bulk repeller molecules (r) on surfaces. (**C**) 2D dissociation. Bond rupture is strongly dependent on the intensity and time-dependence of forces exerted on the bonds. When a bond is broken, the dynamics of anchoring surfaces may influence rebinding.

This theory was widely accepted, although a few discrepancies suggested that some refinements might be needed. The affinity constant, i.e., a measure of the affinity, or thermodynamic stability of TCR-pMHC association (Fig. 1), was reported to be better-correlated with T-cell activation potency than the dissociation rate in some studies. However, since detection sensitivity might be due to the capacity of a single pMHC to engage hundreds of TCRs following the so-called serial triggering mechanism, it could be argued that multiple TCRs might be more efficiently engaged by a pMHC displaying moderate bond lifetime and high association rate, corresponding to a medium affinity constant. In contrast, a pMHC with a very low dissociation rate could not engage many additional TCRs after binding to the first one.

However, subsequent research conducted in a quite different domain cast significant doubt on the validity of this model, paving the way for innovative experimental approaches. Biophysically oriented teams developed highly sensitive methods to monitor the formation and dissociation of single molecular bonds between surface-attached molecules with concomitant control of applied forces (Pierres, 1996). Experiments performed with devices as diverse as atomic force microscopes, laminar flow chambers, optical tweezers, or the biomembrane force probe unambiguously demonstrated that the association of surface-bound molecules could not be accounted for by parameters such as affinity constant, and association or dissociation rates measured in solution (Fig. 1A,B) (Robert et al, 2007). Accordingly, the former interactions were dubbed “2D”, i.e., two-dimensional, in contrast with conventional “3D” parameters. Two specific points were emphasized:

(i) First, the 2D dissociation rate may not be considered as an intrinsic property of a given ligand-receptor pair, since it is strongly dependent on the dynamics of surfaces bearing interacting molecules and particularly the forces exerted on the bond (Fig. 1C). It was first thought that the dissociation rate displayed exponential increase as a function of applied force. However, this was not the whole story: it was rapidly shown that some bonds displayed a fairly counter-intuitive behavior, with a decrease of dissociation rate in response to forces that usually felt in the piconewton range. These bonds were dubbed catch-bonds.
(ii) Another finding that must be borne in mind is that single-bond studies emphasized the multiplicity of binding states displayed by molecular couples, leading to some paradoxical findings such as the dependence of bond dissociation rate on its history or inability of a single constant to account for the kinetics of bond formation. Thus, 2D interactions cannot be fully described by a few well-defined parameters (Fig. 1A–C).

Unsurprisingly, when the TCR-pMHC interaction was studied at the single-molecule level with the aforementioned tools, as reported in two landmark papers (Huang et al, 2010; Huppa et al, 2010), the 2D association and dissociation rates were markedly different from conventional 3D parameters. Indeed, pMHCs with higher T-cell activation potency displayed higher 2D dissociation rates and lower 3D dissociation rates than less active ones (Huang et al, 2010), and it was convincingly shown that the 2D dissociation rate could be modulated by forces exerted by cells (Huppa et al, 2010). In addition, it was later shown that T-cells might generate forces of order of 5 pN within a few seconds. This is interesting because these values match the order of magnitude of TCR-pMHC bond lifetime and resistance to forces. A productive TCR-pMHC interaction might thus generate a pulling force that might break the bond. The relevance of forces to T-cell activation was also supported by the finding that T-cell activation in contact with a ligand-coated surface depends on the stiffness of this surface and the direct demonstration that a mechanical stimulation of the TCR could generate signaling events such as intracellular calcium rises.

The relevance of forces to T-lymphocyte activation was further supported by an important study (Liu et al, 2014). The interaction between OT-I TCR-bearing T-cells and ligand-bearing probes was monitored with single-bond sensitivity together with signaling events, leading to the conclusion that only T-cell-stimulating ligands displayed catch-bond behavior. The idea that activation potency is correlated to a catch-bond behavior was supported in a later study made on 50 oligopeptides (Sibener et al, 2018). While other studies led to the conclusion that the capacity of a pMHC to form catch bonds with cognate TCR was not an absolute requirement for activation (Limozin et al, 2019), forces have been consistently shown to influence TCR sensitivity and antigen discrimination (Fritzsche and Kruse, 2024).

The significance of the proofreading model was also subjected to repeated analysis. The kinetic proofreading model is based on conventional thermodynamic and kinetic reasoning that is relevant to large molecular assemblies. However, if it is considered that T-cell activation may be triggered by a few molecular events, and since bond rupture is a stochastic event, an accurate determination of bond lifetime is expected to require multiple sequential measurements in contradiction with the rapidity of antigen testing. This is the reason why T-cell discriminating power was emphasized to be close to the limitations set by physics principles (Malissen and Bongrand, 2015). The necessity of a balance between TCR accuracy and speed was indeed emphasized.

We feel that three important lessons may be drawn from this article.

First, it reminds us that data quality is an essential part of scientific reasoning. The basis of the paper is indeed a correction of measured 3D parameters of low-affinity TCR-ligand interaction. This point must be kept in mind in a time where the impressive power of data-greedy machine learning algorithms is an incentive to privilege data quantity over quality.

Second, since the OT-I TCR arguably provided the best-studied example of a discrepancy between pMHC stimulation potency and affinity with a given TCR, this report will be an incentive to initiate further studies on the correlation between 3D parameters and activation potency.

Third, while the detailed mechanisms of T-cell activation remain incompletely understood, the development of immunotherapy approaches and important tools such as CAR T-cells generate a need for predictors of immune recognition. A key message of this paper is that 3D affinity is a good candidate for this purpose despite the complexity of mechanisms revealed during the last decade and that might have cast doubt on the validity of this parameter.

In conclusion, the experiments reported in this paper may be viewed as a step toward two complementary goals of T-cell research, namely predicting and explaining T-cell response to antigen challenge.

Indeed, it appears reasonable to hypothesize that a single parameter cannot allow exhaustive prediction of all aspects of the T-cell response to stimulation by a given TCR ligand, including the effect of antigen dose and balance between proliferation and various differentiation pathways, including memory cells, regulators, helpers and effectors. This response is influenced by a number of parameters including the aforementioned resistance to forces but also 2D environmental parameters. It is thus reasonable to speculate that optimal prediction should involve several different parameters. Exhaustive conclusions should thus require a numerical formula or a machine-learning model or both. Arguably, explaining the procedure allowing T-cells to decode the information enclosed in their encounter with TCR ligands and choose an appropriate developmental path will require a suitable combination of experimental approaches currently developed to unravel cell function, including “omic” measuring of biochemical events following stimulation, development of platforms allowing real-time monitoring of cell stimulation with high spatial and temporal resolution, and atomic-scale analysis of molecular response to forces. Due to the complexity of the involved phenomena, it is likely that the processing of multimodal results will require a recourse to artificial intelligence models that was met with impressive success in biomedical research.
